# Awareness, Views, Perceptions, and Beliefs of Pharmacy Interns Regarding Digital Health in Saudi Arabia: Cross-sectional Study

**DOI:** 10.2196/31149

**Published:** 2021-09-03

**Authors:** Saud Alsahali

**Affiliations:** 1 Department of Pharmacy Practice Unaizah College of Pharmacy Qassim University Qassim Saudi Arabia

**Keywords:** digital health, eHealth, mHealth, telehealth, telemedicine, attitude, awareness, pharmacy interns

## Abstract

**Background:**

Digital health technologies and apps are rapidly advancing in recent years. It is expected to have more roles in transforming the health care system in this era of digital services. However, limited research is available regarding delivering digital health education in pharmacy and the pharmacy students’ perspectives on digital health.

**Objective:**

This study aims to assess pharmacy interns’ awareness of digital health apps in Saudi Arabia and their views regarding the coverage of digital health in the education of pharmacists. In addition, we assessed the interns’ perceptions and beliefs about the concepts, benefits, and implementation of digital health in practice settings.

**Methods:**

A cross-sectional study using a web-based survey was conducted among pharmacy interns at Unaizah College of Pharmacy, Qassim University, Saudi Arabia. An invitation with a link to the web-based survey was sent to all interns registered at the college between January and March 2021.

**Results:**

A total of 68 out of 77 interns registered in the internship year participated in this study, giving a response rate of 88%. The mean total score for pharmacy interns’ awareness of digital health apps in Saudi Arabia was 5.66 (SD 1.74; maximum attainable score=7). The awareness with different apps ranged from 97% (66/68) for the Tawakkalna app to 65% (44/68) for the Ministry of Health 937 call center. The mean total score for attitude and beliefs toward concepts and benefits of telehealth and telemedicine apps was 58.25 (SD 10.44; maximum attainable score=75). In this regard, 84% (57/68) of the interns believed that telehealth could enhance the quality of care, 71% (48/68) believed that it could help effectively provide patient counseling, and 69% (47/68) believed it could improve patients’ adherence to therapy. In this study, 41% (28/68) believed that the current coverage of digital health in the curriculum was average, whereas only 18% (12/68) believed it was high or very high coverage. Moreover, only 38% (26/68) attended additional educational activities related to digital health. Consequently, the majority (43/68, 63%) were of the opinion that there is a high or very high need to educate and train pharmacists in the field of digital health.

**Conclusions:**

Overall, the interns showed good awareness of common digital health apps in Saudi Arabia. Moreover, the majority of the interns had positive perceptions and beliefs about the concepts, benefits, and implementation of digital health. However, the findings showed that there is still scope for improvement in some areas. Moreover, most interns indicated that there is a need for more education and training in the field of digital health. Consequently, early exposure to content related to digital health and pharmacy informatics is an important step to help in the wide use of these technologies in the graduates’ future careers.

## Introduction

### Background

The use of technology to deliver health care services and health education has grown rapidly in recent decades. Moreover, the digital transformation of health care is gaining more attention with the recent major developments in information and telecommunication technologies, the Internet of Things, virtual care, remote monitoring, artificial intelligence, big data analytics, and digital platforms [1]. Digital health is defined by the World Health Organization as “the field of knowledge and practice associated with the development and use of digital technologies to improve health.” It is a broad term and includes mobile health (mHealth), eHealth, telehealth, telemedicine, and other artificial intelligence applications in health care [1,2].

Digital health technologies have the potential to improve health outcomes, improve the efficiency of health systems, empower patients with knowledge, improve access to health care, and lead to cost savings. For example, telehealth, which involves the use of virtual technology to provide health information, awareness, consultation, monitoring, and many other forms of medical care, helps to improve access to health care while maintaining health expenses at a reasonable level. Moreover, in telemedicine, technology is used to deliver clinical practice services in a distance setting [3,4]. In telemedicine, health care providers can use video conferencing and other technology apps to exchange health information in clinical practice and interpret lab results. Telemedicine serves as an appropriate alternative for in-person visits to health care providers’ offices. It can help to decrease the impact of physician workforce shortages and the lack of specialized care in some geographic areas [5]. Thus, patients can easily receive health care at acceptable costs and effective services [6].

It is evident in the literature with many studies from different countries that telehealth and telemedicine are effective tools for patient care, including for people with chronic diseases such as diabetes and mental health, and can provide critical care services for distant areas [7-9]. Moreover, digital health, including telehealth, plays a pivotal role during pandemics, disasters, and emergencies [10,11]. For example, during the COVID-19 pandemic, to decrease the transmission of the virus, many countries have implemented lockdowns and expanded the use of technology for many services, especially health care services and education. The acceleration of technology adoption to deliver health services during the pandemic provided physicians with opportunities to manage their patients and work with the latest technology to have safe and effective options to access health care services [11].

The global market size of telemedicine in 2019 was estimated at US $27.8 billion, with a promising growth rate in the next few years [12]. Telehealth expansion will require substantial restructuring of laws and regulations to protect both patients and providers [13]. Many governments and private health systems have invested in introducing telehealth services. Some have well-integrated networks, such as the Ontario Telemedicine Networks in Canada and telehealth services in Veteran Affairs in the United States [14]. The Australian government has encouraged the use of telehealth in medical consultations and introduced reimbursement for some services, including video-based consultations [14,15].

In Saudi Arabia, the initiatives to adopt eHealth and telehealth services date back to more than three decades. For example, the Center of eHealth was established at the King Faisal Specialist Hospital & Research Centre in Riyadh, which is considered a tertiary hospital and one of the leading institutions in the Middle East. The center has international cooperation via videoconferencing with other centers around the world, and it has telemedicine network centers distributed in many regions in Saudi Arabia to deliver health care and consultation to distant areas [16]. Recently, in 2017, as part of Saudi Arabia’s Vision 2030, the implementation of the digital transformation plan for the public and private health sectors began. Consequently, the Saudi Ministry of Health (MOH) has created many mobile apps to facilitate administrative processes for patients and to allow them to obtain medical consultations and refill their medications [17]. For example, the Saudi MOH introduced *Mawid*, which acts as a national platform to book medical appointments and to manage referrals from primary care centers to specialized centers [17]. In 2019, the Saudi MOH launched *Sehhaty*, which provided personal health information and improved knowledge about public health and healthy lifestyles; it was subsequently used to book COVID-19 vaccine appointments [16]. Moreover, the Saudi MOH introduced a call center (937) that received calls and offered answer services for the patients and clients for any medical questions related to symptoms or medications [17]. During the COVID-19 pandemic, the government launched several mobile apps to prevent the spread of COVID-19. The government introduced *Tetamman* to guide and help people who were under isolation because of contact with infected persons or those returning from abroad. The *Tawakkalna* app was used to provide movement permission during curfew times and as electronic personal identification that included all national documents and provided information on the infection status of people, allowing them to enter restaurants, supermarkets, and governmental authorities. In August 2020, an app named *Tabaud* was used for contact tracing of infected cases [17].

As digital health grows rapidly with massive investment from the government of Saudi Arabia in line with Saudi Vision 2030, it is important to ensure all challenges and barriers for the wide adoption and use of digital health are addressed. The barriers reported in the literature include that digital health technologies and telemedicine could be perceived as technically challenging for some health care professionals [18]. In addition, it is of great importance to ensure that health care professionals are aware of the economic and clinical values of digital health technologies, to increase their acceptance of an effective method to deliver health care, and to ensure they have the necessary skills to use it in their daily work and teach patients how to access telehealth efficiently [18]. In addition, there is a good opportunity to cover the use of digital health in the curriculum of medical and pharmacy colleges. The earlier coverage of digital health in the initial education and training of health care professionals during their university studies has the potential to increase their comfort and familiarity with the use of the various digital health technologies, leading to wide and rapid use in their future practice and to help promote the adoption of this technology among the community as well [5].

### Objectives

The objectives of this study are to assess the pharmacy interns’ awareness of common digital health apps in Saudi Arabia and to assess their views regarding the need for the coverage of digital health in the education and training of pharmacists. Moreover, the study assessed the interns’ perceptions and beliefs about the concepts and benefits of digital health and their beliefs regarding the implementation of telehealth in practice settings.

## Methods

### Study Design and Setting

This was a cross-sectional study that used a web-based survey. The target population of this study were PharmD interns at the Unaizah College of Pharmacy, Qassim University, Saudi Arabia. The PharmD interns were those who completed all the didactic curriculum (ie, 5 years) and were enrolled in the sixth year of the program (ie, the internship year). During the study period, the PharmD interns had already spent more than 6 months of training and clinical rotations in the hospital setting. All 76 PharmD interns were invited to participate in the study. Ethical approval was obtained from the Health Research Ethics Committee at Qassim University, Saudi Arabia (reference number 20-06-12).

### Development and Administration of the Questionnaire

The questionnaire used in this study was developed based on previous studies [16,19-21]. The final questionnaire consisted of four parts. The first part assessed the interns’ awareness of the digital health apps in Saudi Arabia. It examined whether the interns were aware of the seven common mHealth apps that are used in Saudi Arabia. The total awareness was calculated by giving 1 point if the intern was aware of the app and 0 if the intern was not aware. Consequently, the attainable score ranged from 0-7 points. The second part consisted of five questions that focused mainly on the interns’ views regarding the need to cover digital health in the education and training of pharmacists. The interns were asked whether they attended any training course, conference, or educational activities related to digital health (the answer choices were yes or no). For the four remaining questions of this part, the interns were asked about their opinion regarding the current coverage of digital health in their PharmD program, the importance of education and training for pharmacists in the field of digital health, and their familiarity with pharmacy informatics in their practice setting. The interns were given a choice to express their views and opinions on a 5-point scale from very low (1) to very high (5). For the sake of comparison between male and female interns, the total score was calculated with a maximum score of 20.

The third part of the questionnaire consisted of 15 statements that assessed the perceptions and beliefs of pharmacy interns about the adoption of digital health, including telehealth and telemedicine in the health care system and its related benefits*.* It focused on the usability of telehealth apps in their work and the ability of these apps to support clinical decisions and facilitate good clinical practice. Moreover, it included statements to assess the beliefs of interns about whether digital health can provide psychological support for patients and whether it can be used effectively in patient counseling and can enhance patients’ adherence and access to health services. The answers were rated on a 5-point Likert scale ranging from strongly disagree (1) to strongly agree (5). The attainable scores ranged from 15-75 points. The fourth part of the questionnaire assessed interns’ beliefs regarding the implementation and complexity of telehealth and telemedicine and consisted of six statements. The first three questions focused on implementation and were measured on a 5-point Likert scale ranging from *strongly agree* (5 points) to *strongly disagree* (1 point). For the remaining three statements that focused on the complexity of telemedicine, the Likert scale score was reversed and graded using 5 points for *strongly disagree* and 1 point for *strongly agree* as they presented negative views. The attainable scores ranged from 6-30 points.

To ensure the validity and applicability of the questionnaire in our setting, it was sent to 2 reviewers with expertise in both digital health and questionnaire-based studies to comment and provide feedback on the questionnaire. Their comments and feedback were incorporated, and minor modifications were made. Then, it was given to 3 interns to check the clarity, applicability, and suitability; then, the questionnaire was finalized and made ready for web-based distribution.

The questionnaire was distributed on the internet via WhatsApp (Facebook Inc), and all interns were invited to participate in the study. The interns were provided with a brief overview of the survey, including its aim and the fact that their participation was voluntary, and they could withdraw at any time during the study.

### Analysis of the Data

SPSS version 20.0 (IBM Corp) was used to analyze the data and to summarize the responses of interns. Descriptive statistics, which included frequencies and percentages, were used to summarize the responses of interns to the survey questions. Inferential statistics (ie, Student two-tailed *t* test) were used to examine whether there were significant differences in the mean scores between males and females. Statistical significance was set at *P*<.05.

## Results

### Demographic Data

Of the 77 interns, 68 completed the survey, giving a response rate of 88%. In terms of gender, 29% (20/68) were male and 71% (48/68) were female. In terms of age, the mean age (SD) was 23.68 years (SD 0.87), ranging from 23 to 26 years.

### Pharmacy Interns’ Awareness of Digital Health Apps in Saudi Arabia

Overall, the mean total score for pharmacy interns’ awareness of mHealth apps in Saudi Arabia was 5.66 (SD 1.74; maximum attainable score=7). As shown in Table 1, almost all interns 97% (66/68) were aware of the Tawakkalna app. In addition, most of the interns were aware of other apps, including Sehhaty 88% (60/68), Mawid 88% (60/68), Tabaud 79% (54/68), and Wasfaty 75% (51/68). However, only 65% (44/68) of the interns were aware of the 937 call center services provided by the Saudi MOH. There was no statistically significant difference in the mean score of pharmacy interns’ awareness of digital health apps between male interns of 5.45 (SD 1.98) and female interns of 5.75 (SD 1.65; *P*=.52).

**Table 1:** Awareness of pharmacy interns of digital health apps in Saudi Arabia (N=68).

Digital health apps	Yes, n (%)	No, n (%)
Sehhaty	60 (88)	8 (12)
Mawid	60 (88)	8 (12)
Wasfaty	51 (75)	17 (25)
Tawakkalna	66 (97)	2 (3)
Tabaud	54 (79)	14 (21)
Tetamman	52 (77)	16 (24)
Saudi MOH^a^ 937 call center	44 (65)	24 (35)

^a^MOH: Ministry of Health.

### Pharmacy Interns’ Views Regarding the Need for the Coverage of Digital Health in the Education and Training of Pharmacists

In this study, 38% (26/68) of the interns participated in additional educational activities or training courses on telehealth and eHealth. Among the interns, 41% (28/68) believed that the current coverage of telehealth and telemedicine was average, while only 18% (12/68) believed it was high or very high coverage. Furthermore, 63% (43/68) of interns were of the opinion that there is a high or very high need to educate and train pharmacists to be able to use digital health apps in their practice. Furthermore, more than two-thirds 68% (46/68) were of the opinion that training on the use of pharmacy informatics and digital health was necessary for the internship year (high or very high need), as shown in Table 2. There was no statistically significant difference in the mean score of responses of pharmacy interns regarding the need for the coverage of digital health in the education and training of pharmacists between male interns of 14.95 (SD 1.90) and female interns of 14.50 (SD 3.18; *P=*.56; maximum score for the four statements is 20).

**Table 2:** Views of pharmacy interns regarding the need for coverage of the digital health in the education and training of pharmacists.

Question	Pharmacy interns (n=68), n (%)				
	Very low	Low	Average	High	Very high
What do you think of the current coverage of telehealth and digital health in the PharmD program?	6 (9)	22 (32)	28(41)	10 (15)	2 (3)
To what extent is training in the use of telehealth necessary for pharmacists?	1 (1)	4 (6)	20 (29)	24 (35)	19 (28)
To what extent you are familiar with electronic health and drug information apps and databases (eg, UpToDate)?	1 (1)	8 (12)	17 (25)	19 (28)	23 (34)
To what extent do you believe the need for training in the use of telehealth apps and pharmacy informatics is necessary for the internship year program?	2 (3)	4 (6)	16 (24)	26 (38)	20 (29)

### Pharmacy Interns’ Perceptions and Beliefs About Telehealth and Telemedicine

In this study, 72% (49/68) of interns agreed that telehealth could help reduce medical errors, and 84% (57/68) agreed that telehealth could enhance the quality of care. In addition, 75% (51/68) of participants believed that telehealth and telemedicine could reduce the number of physical visits, and 65% (44/68) agreed that they could overcome the inconvenience of going to a physician or a pharmacist. Moreover, 72% (49/68) of participants agreed that telehealth can enable pharmacists to accomplish tasks more quickly, and 63% (43/68) believed that telehealth can improve clinical decisions. In terms of patient education and counseling, 71% (48/68) of participants believed that telehealth and telemedicine can help provide effective patient counseling, and 69% (47/68) believed that telehealth apps can improve the adherence to therapy of patients, as shown in Table 3. Overall, the mean total score for attitude and beliefs toward telehealth apps and telemedicine was 58.25 (SD 10.44; maximum attainable score=75), with no statistically significant difference in the mean score between male interns of 59.8 (SD 8.25) and female interns of 57.6 (SD 11.78; *P*=.45).

**Table 3:** Perceptions and beliefs of pharmacy interns regarding telehealth and telemedicine.

Statement	Pharmacy interns (n=68), n (%)				
	Strongly agree	Agree	Neutral	Disagree	Strongly disagree
Telehealth can reduce medical errors.	32(47)	17 (25)	14 (21)	4 (6)	1 (1)
Telehealth can enhance the quality of patient care.	29 (43)	28 (41)	8 (12)	1 (1)	2 (3)
Telehealth can facilitate diagnosis and treatment.	12 (18)	23 (34)	19 (28)	10 (15)	4 (6)
Telehealth can increase communication among health care providers.	26 (38)	23 (34)	15 (22)	2 (3)	2 (3)
Telehealth can reduce the number of physical visits to health care centers.	23 (34)	28 (41)	13 (19)	3 (4)	1 (1)
Telehealth can enable pharmacists to accomplish tasks more quickly.	23 (34)	26 (38)	16 (24)	2 (3)	1 (1)
Telehealth can improve clinical decisions.	17 (25)	26 (38)	16 (24)	7(10)	2 (3)
Telehealth can provide more comprehensive health care services.	19 (28)	29 (43)	16 (24)	2 (3)	2 (3)
Telehealth is convenient and can overcome the inconvenience of going to a physician or a pharmacist.	14 (21)	30 (44)	18 (26)	5 (7)	1 (1)
Psychological support to patients can be provided effectively through telehealth.	14 (21)	23 (34)	22 (32)	7 (10)	2 (3)
Health education and patient counseling can be provided effectively through telehealth.	25 (37)	23 (34)	17 (25)	2 (3)	1 (1)
Virtual consultations allow prompt interventions.	11(16)	33 (49)	19 (28)	3 (4)	2 (3)
Telehealth can help in saving time.	24 (35)	30 (44)	7 (10)	3 (4)	4 (6)
Telehealth can enhance access to health care services.	24 (35)	30 (44)	7 (10)	2 (3)	5 (7)
Telehealth and electronic apps can improve adherence to therapy of patients.	19 (28)	28 (41)	16 (24)	3 (4)	2 (3)

### Pharmacy Interns’ Beliefs Regarding the Implementation and Complexity of Telehealth

More than two-thirds (48/68, 71%) of the participants believed that telehealth apps are compatible with pharmacists’ duties, 56% (38/68) reported that they fit well with the way they liked to work, and 74% (50/68) thought that telehealth apps could be implemented through several devices and digital platforms. Regarding complexity, 35% (24/68) disagreed that digital health and telehealth required a lot of mental effort, whereas 34% (23/68) were neutral toward this statement. In this study, 46% (31/68) thought that digital health and telemedicine could increase workload, and 54% (37/68) reported that it could threaten patient privacy, as shown in Table 4. Overall, the mean total score for pharmacy interns’ views regarding the implementation and complexity of telehealth use was 19.77 (SD 3.16; maximum attainable score=30). There was no statistically significant difference in the mean score of views regarding the implementation and complexity of telehealth use between male interns of 19 (SD 3.07) and female interns of 20.10 (SD 3.17; *P=*.19).

**Table 4:** Beliefs of pharmacy interns regarding the implementation and complexity of telehealth.

Question	Pharmacy interns (n=68), n (%)				
	Strongly agree	Agree	Neutral	Disagree	Strongly disagree
I believe telehealth is compatible with the professional duties of pharmacists.	9 (13)	39 (57)	15 (22)	3 (4)	2 (3)
I think telehealth fits well with the way I like to work.	13 (19)	25 (37)	19 (28)	9 (13)	2 (3)
I think telehealth can be implemented through several devices and digital platforms.	16 (24)	34 (50)	8 (12)	5 (7)	5 (7)
I believe using telehealth requires a lot of mental effort.	3 (4)	18 (26)	23 (34)	12 (18)	12 (18)
I think telehealth increases staff workload.	9 (13)	22 (32)	12 (18)	18 (26)	7 (10)
I think telehealth threatens information confidentiality and patient privacy.	9 (13)	28 (41)	14 (21)	10 (15)	7 (10)

## Discussion

### Principal Findings

Digital health technologies and apps are rapidly advancing and have gained importance as they play a vital role in facilitating access to health care. They have multiple features that can save time for patients and clinicians in a low-cost and convenient manner. Moreover, telehealth has gained more importance in the education and training of health care professionals in recent years [22]. This study assessed pharmacy interns’ awareness of digital health and their views about adopting this technology and its usability for their work. The interns showed good understanding of common apps used in Saudi Arabia. Approximately all participants were aware of the Tawakkalna app, which represents the highest percentage among the apps. In May 2021, the Saudi press agency reported that the Tawakkalna app had a high number of users, reaching more than 20 million users in Saudi Arabia, which is considered the highest number of users among all telehealth apps. This app was developed to show the health status or users and is required to enter markets, public, or governmental buildings [23]. In addition, it is a GPS-enabled app, which is used to control and limit the movement or residents during the curfew time implemented in the COVID-19 pandemic. In addition, it is used to issue permissions for exceptional situations to move during the curfew time. Moreover, it is connected with another app, *Tabaud*, which sends alerts to the users of the app to inform them that they are in close contact with confirmed cases of COVID-19 [24].

The interns showed positive views regarding the inclusion of digital health in the education and training of pharmacists, with the majority being aware of the current drug information apps and digital databases. As reported in previous studies conducted across different communities, the participants in this study showed interest in telehealth. They reported that it was necessary for their education and training and that it provided knowledge and opportunities to develop their skills, which could encourage students to use eHealth techniques in the future [25,26]. Although the interns showed good awareness of digital health apps and positive views regarding the inclusion of digital health technology in their education and training, many of them did not attend additional training in telehealth. Consequently, many believed that telehealth coverage in their PharmD program needs to be increased. Only 38% (26/68) of the interns attended training or workshops on digital health. In addition, 41% (28/68) believed that digital health and pharmacy informatics coverage in their PharmD program was low or very low. These findings are similar to those reported by a previous study conducted at the end of 2020 among medical students. The study concluded that only 17.4% of medical students had prior exposure to telehealth despite having a high level of interest in using telehealth in the future [26].

In the literature, there is a very limited number of studies related to the delivery of digital health education and training [14]. As reported by Edirippulige and Arm field [14], there are two main types of telehealth-related education and training. The first type is a traditional university course, whereas the second is continuing professional development, which focuses on professional skills [14]. As digital health has gained more importance in recent years, more education and training opportunities were recently integrated into our PharmD curriculum at the Unaizah College of Pharmacy, Qassim University. These include adding and integrating more topics related to digital health, pharmacy informatics, and automation in some pharmacy practice courses. In addition, an elective course in pharmacy informatics was added for additional training. Moreover, further opportunities were made available in the internship year for training in digital health, including pharmacy automation and digital drug information resources. This is particularly important for increasing knowledge and acceptance of this technology of the students. Several studies have indicated that telemedicine adoption is affected by the knowledge and perceptions of health care providers [20,27]. Other studies have shown that early exposure to telehealth and telemedicine practices early in health care education greatly impacts the knowledge and views of providers regarding their use in future work [25,26,28,29].

Most students showed positive perceptions and beliefs regarding telehealth apps. Approximately 72% (49/68) of interns agreed or strongly agreed that telehealth and telemedicine could help reduce medication errors compared with nearly 69.5% of health care professionals in an Ethiopian study [21]. In addition, 75% (51/68) of respondents believed that telemedicine reduces the need for physical visits, compared with 76.2% of respondents in the Ethiopian study [23]. Approximately 69% (47/68) of interns believed that telehealth apps help improve patient adherence to treatment, which is comparable with the findings of a study conducted among health care professionals and medical students in Saudi Arabia that reported that patient adherence might be improved with the help of technology [30]. A total of 65% (44/68) of interns agreed or strongly agreed that telehealth apps are convenient and can overcome the inconvenience of attending physicians or pharmacists, which is comparable with the 65.2% reported by Peprah et al [31] among university students.

The majority of interns showed positive views about the implementation of telehealth and indicated that telehealth is compatible with their professional duties. However, 31% (21/68) of interns agreed or strongly agreed that digital health requires more mental effort, and 46% (31/68) believed that telehealth could increase their workload. The findings related to the complexity of telehealth use in this study are consistent with the findings reported in previous studies [21,30]. A study conducted in Saudi Arabia by Thapa et al [30] reported that students perceived that the use of eHealth would increase work-related stress and could delay responses to patients’ needs. In addition, this study found that more than half of the interns 54% (37/68) believed that telehealth might threaten the information privacy of patients, compared with 66% reported by Birukand Abetu [21]. The privacy of patient information is generally one of the challenges that has been assessed in many previous studies and should be considered when adopting telehealth services [32,33]. Many recent studies have revealed that data protection regulations are among the critical factors limiting the adoption of virtual software apps used in remote health care [34]. Easy accessibility and sharing of information may raise concerns regarding data confidentiality and misuse. However, in Saudi Arabia, huge investments have been made to ensure data protection and cyber security. In addition, through the National Health Information Center, it is required that all telemedicine practices follow Saudi Health Information Exchange Policies, which are well secured and highly consider patient rights and health information privacy [17,34].

### Strengths and Limitations

This is one of the few studies in the literature that explored digital health from the perspective of pharmacy interns. In addition, a high percentage of the target population responded to the survey (68/77, 88%). However, this study has some limitations. First, it was conducted at one pharmacy college in Saudi Arabia; therefore, the findings might not be generalizable to other institutions in Saudi Arabia. However, given the limited literature in this field, we believe that this study provides useful insights and guidance for educators and policymakers in pharmacy education.

### Conclusions

The use of digital health has gained importance and is expected to have greater roles today and in the future. It can help patients and clinicians in a low-cost and convenient manner to provide an acceptable level of health care. In addition, digital health can support clinical decisions through consultations and the exchange of information and experiences through technology. Telehealth and telemedicine can help in making health care more accessible to remote areas and during pandemic situations. Overall, the interns showed a good awareness of common digital health apps in Saudi Arabia. In addition, the majority of the interns had positive perceptions and beliefs about the concepts, benefits, and implementation of digital health. However, the findings showed that there is still scope for improvement in some areas. Moreover, most interns indicated that there is a need for more education and training in the field of digital health and pharmacy informatics. Consequently, early exposure to content related to digital health and pharmacy informatics is an important step to help in the wide use and apps of these technologies in the future careers and practices of graduates.

## Abbreviations

mHealth: mobile health
MOH: Ministry of Health
